# Decoding Rich Spatial Information with High Temporal Resolution

**DOI:** 10.1016/j.tics.2015.08.016

**Published:** 2015-11

**Authors:** Mark G. Stokes, Michael J. Wolff, Eelke Spaak

**Affiliations:** 1 Department of Experimental Psychology, University of Oxford, Oxford, UK; 2 Oxford Centre for Human Brain Activity, University of Oxford, Oxford, UK; 3 Department of Experimental Psychology, University of Groningen, Groningen, The Netherlands

**Keywords:** Neural decoding, multivariate pattern analysis, orientation tuning, magnetoencephalography, electroencephalography, spatiotemporal information

## Abstract

New research suggests that magnetoencephalography (MEG) contains rich spatial information for decoding neural states. Even small differences in the angle of neighbouring dipoles generate subtle, but statistically separable field patterns. This implies MEG (and electroencephalography: EEG) is ideal for decoding neural states with high-temporal resolution in the human brain.

A major challenge in cognitive neuroscience is to discriminate brain states with high spatial and temporal resolution. These two dimensions are often considered mutually exclusive for non-invasive human studies. Functional magnetic resonance imaging (*fMRI*) can resolve detailed spatial patterns of activity, but has notoriously poor temporal resolution; whereas methods that track electrical activity provide rich temporal information, but lack spatial precision. However, a recent paper by Cichy *et al*. invites us to re-evaluate this classic dichotomy. Using a combination of empirical data and theoretical modelling, they argue that the signals measured with magnetoencephalography (MEG) actually contain rich spatial information that can be used to differentiate extremely subtle neural states [1]. This could be a game changer for high-temporal resolution methodologies that have been long considered too coarse for differentiating fine-scale neural coding.

Just over a decade ago, fMRI experienced a major breakthrough inspired by a relatively simple idea: idiosyncratic patterns of activity carry important information. The test case was orientation decoding. It turns out that activity patterns in visual cortex can reliably predict the orientation of a grating stimulus presented to the subject (e.g., [2]). The general importance of this finding lies in its broader implication. Different orientations are not represented in different brain areas, but within narrow cortical columns that are distributed throughout the retinotopic landscape of visual cortex. Therefore, if it is possible to decode the orientation of a grating stimulus in visual cortex, perhaps it is also possible to decode other distributed, and spatially overlapping neural states, and in other brain areas. Indeed, perhaps patterns of fMRI activity could even carry informational content comparable to the gold standard single unit recordings in non-human primates [3].

The key insight for the fMRI community was that subtle biases in the distribution of neurons tuned to one feature or another could lead to subtle differences in the activity of a sampled voxel (schematised in Figure 1A). Although such biases would be weak, they could be pooled together over a number of samples (i.e., voxels) to statistically differentiate activity patterns. This approach has come to be known as multivariate pattern analysis (MVPA) [4], and has changed the way people think about fMRI. Decoding overlapping population codes for orientation encouraged the field to think more about information coded in a pattern of activity rather than differences in mean activity in certain brain areas [5].

As orientation decoding was the test-ground for fine-scale pattern decoding in fMRI, Cichy *et al.* set out to show that MEG could also be used to decode spatially overlapping neural states. Other studies have shown that orientation information can be decoded from the visual evoked response in MEG and EEG using multivariate pattern analysis 6, 7. However, there are a number of possible confounds that were raised in the fMRI literature that could potentially explain orientation decoding based on coarse spatial differences (e.g., coarse-scale activity differences due to the over-representation of cells tuned to particular orientations; [8]). Cichy and colleagues systematically address a large number of such possible confounds, concluding each time that MEG is able to decode genuine information about the orientation of presented stimuli. The authors concede that it is impossible to claim that their efforts were exhaustive. Indeed, just like the fMRI debate, it is likely that other potential explanations will surface, and would need to be addressed in future studies. Notwithstanding this caveat, Cichy *et al.* present an impressive set of experiments all seemingly pointing to an important conclusion: MEG can resolve spatially overlapping representations.

As reviewed above, previous fMRI studies argued that orientation decoding is driven by subtle differences in sampling small-scale biases in the distribution of tuned cells. However, the spatial resolution of MEG is far coarser than fMRI. So what is the mechanism that could explain genuine orientation decoding? Cichy *et al*. propose a surprisingly simple idea (schematised in Figure 1B).

It is well-established that electrical activity in aligned cells generates a dipole which projects to the scalp surface. EEG measures the electric potential at the scalp surface, whereas MEG measures the magnetic field. The spatial distribution of the field depends on the location of the dipole, but critically, also on its angle. Cichy and colleagues argue that because the surface of the cortex is irregular, even dipoles from neighbouring clusters of cells will have different angles, resulting in separable field patterns at the scalp surface (see Figure 1B i). Although these patterns will be idiosyncratic to a given subject (depending on subtle differences in cortical folding), systematic differences within participants can be differentiated using multivariate classification. So exactly like MVPA for fMRI, it should be possible to differentiate spatially overlapping brain states by analysing subject-specific patterns (see Figure 1B ii), even though group differences would typically just average out.

If MEG/EEG can be a source of such rich spatial information, then why are these non-invasive methods so often considered to have poor spatial resolution? The classic problem limiting spatial resolution in MEG/EEG is source ambiguity. Strictly, it is not possible to localise with certainty the source of the field measured at the scalp surface. There is no unique solution, but theoretically infinitely many solutions that could generate the same pattern of observed activity. To reverse engineer the location of the source from the observed scalp distribution runs up against the obstinate inverse problem. Although sophisticated methods have been developed to constrain probabilistic solutions (e.g., [9]), the inherent uncertainty results in a relatively coarse estimate of the underlying source. However, if the purpose of the analysis is to track differential brain states over time, rather than localise activity differences, then the inherent ambiguity hardly matters.

We predict that multivariate decoding will revolutionise MEG/EEG just as it did fMRI. The key insight is that these measures contain rich spatial information, even if the source localisation is inherently ambiguous. As the fMRI community has moved from localising blobs of condition-specific differences to measuring information coded in activity patterns, so the MEG/EEG community will embrace MVPA for decoding neural states. Moreover, coupled with the exquisite temporal resolution inherent to electromagnetic measures of brain activity, MEG/EEG could really become the method of choice for exploring the spatiotemporal dynamics of human brain activity.

## Figures and Tables

**Figure 1:**
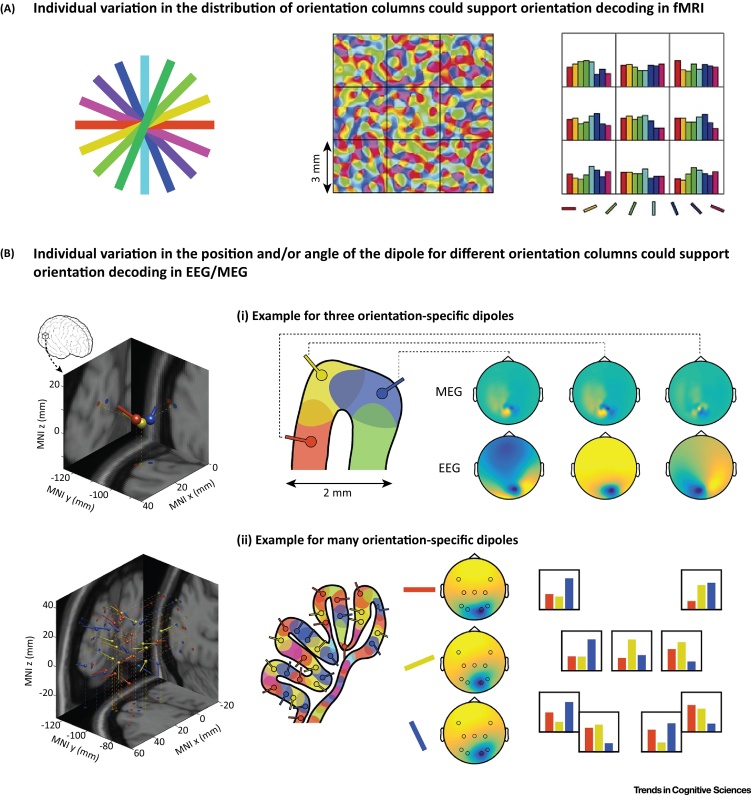
The Proposed Basis for Decoding Stimulus Orientations in EEG and MEG is Analogous to fMRI. (A) Orientation-selective neurons are clustered to form orientation-specific cortical columns in the visual system (orientation-preference coloured coded in the middle panel). Although fMRI cannot resolve activity from specific columns, each voxel samples an uneven distribution of columns resulting in weak but reliable voxel-wise preferences. Stimulus orientation can be decoded from the ensemble pattern of subtle preferences across a number of voxels (9 voxels schematised here; figure adapted from [10]). (B) A similar principle could explain orientation decoding with MEG/EEG. Variation in the angle of neighbouring dipoles could generate separable signals at the scalp surface. i. For example, three dipoles approximately 2 mm apart but with very different angles result in easily distinguishable MEG (upper row) and EEG (lower row) topographies. ii. Such differences will tend to average out with increasing numbers of dipoles (e.g., thirty dipoles, each tuned to one of three stimulus orientations, distributed along visual cortex generate very similar field topographies). However, just like decoding with fMRI, multivariate pattern analysis can differentiate stimulus-orientation by pooling orientation-specific information contained in the subtle biases for each sensor.
